# Multiple Vehicle Cooperative Localization with Spatial Registration Based on a Probability Hypothesis Density Filter

**DOI:** 10.3390/s140100995

**Published:** 2014-01-08

**Authors:** Feihu Zhang, Christian Buckl, Alois Knoll

**Affiliations:** 1 Robotics and Embedded Systems, Technische Universität München, Garching bei München, Germany; E-Mail: knoll@in.tum.de; 2 Fortiss GmbH, Guerickestr. 25, München 80805, Germany; E-Mail: buckl@fortiss.org

**Keywords:** random finite set, PHD filter, spatial registration

## Abstract

This paper studies the problem of multiple vehicle cooperative localization with spatial registration in the formulation of the probability hypothesis density (PHD) filter. Assuming vehicles are equipped with proprioceptive and exteroceptive sensors (with biases) to cooperatively localize positions, a simultaneous solution for joint spatial registration and state estimation is proposed. For this, we rely on the sequential Monte Carlo implementation of the PHD filtering. Compared to other methods, the concept of multiple vehicle cooperative localization with spatial registration is first proposed under Random Finite Set Theory. In addition, the proposed solution also addresses the challenges for multiple vehicle cooperative localization, e.g., the communication bandwidth issue and data association uncertainty. The simulation result demonstrates its reliability and feasibility in large-scale environments.

## 1. Introduction

Accurate vehicle localization is the current trend in the field of intelligent vehicles for the purpose of autonomous driving. Single vehicle localization is often performed by fusing both proprioceptive and exteroceptive sensors presented in [1]. Furthermore, GPS [2,3], cameras [4], laser scanners [5,6], dead reckoning [7] and Boolean information [8] are also used. However, with the developed inter-vehicle communication technology [9], the information exchange among the vehicles becomes available. With a wireless communication device, the multiple vehicle cooperative localization can be improved by taking advantage of information sharing. Various methods are investigated for cooperative localization, such as Extended Kalman Filter [10–12], Bayesian formalism [13], Markov Localization [14], Maximum Likelihood Estimation [15] and Maximum *A Posteriori* Estimation [16,17]. In our previous work [18], we presented a probability hypothesis density (PHD) solution for the purpose of addressing the following issues:

- The communication bandwidth issue.
  The communication bandwidth issue is introduced by Nerurkar [19,20]. The wireless transceivers used for information exchanging may have a low bandwidth, due to the restriction of the power. However, a high communication bandwidth for transmitting the estimation state, including large covariance matrices, is often required by the standard fusion methods.
- The uncertainty issue.
  Data association plays an important role in the cooperative localization introduced by Franchi *et al.* [21,22]. In their work, a data associator is proposed to compute all possible hypotheses based on the distances. With the development of the V2V (vehicle-to-vehicle) communication systems, vehicles are able to localize and identify the other members of the group correctly. Various aspects of inter-vehicle communication are surveyed in [23]. However, measurements often consist of clutters, which are hard to identify when aggregated in a small region. The standard techniques that are based on nearest neighbor association are therefore invalidated. In order to build up a more generic localization framework, addressing the association uncertainty is required.

Based on the above issues, we proposed the PHD filter to consider the whole group behavior instead of each vehicle [18]. In the PHD filter, the collection of individual vehicles is treated as a set-valued state, and the collection of individual measurements is treated as set-valued observation. Modeling set-valued state and set-valued observation as a random finite set (RFS) provides a novel potential in multiple vehicle cooperative localization, which has already been exhibited previously.

This paper enhances the previous work by joint spatial registration and states estimation simultaneously. Spatial registration is considered as follows: once the measurement is acquired form the sensor, two kinds of errors are also included. One belongs to the random noises described as Gaussian white noise, while the other is called the bias (systematic error), which is considered as a fixed value originated from the sensor calibration. The purpose of the spatial registration is to estimate the related bias during the whole process. To the best of the authors' knowledge, the spatial misregistration problem has not been considered in multiple vehicle cooperative localization under Random Finite Set Theory. The multiple vehicle states and the biases of the sensors are jointly estimated recursively via the PHD filter. The sequential Monte Carlo (SMC) method is used to implement the approach, considering non-linear and non-Gaussian conditions [24].

In this paper, we adopt the same framework as proposed previously: the measurements from both proprioceptive and exteroceptive sensors are projected to a global plane that constitutes of the observation set. The PHD filter recursively estimates the dynamic states and spatial biases according to the observations.

The contributions of the proposed approach are as follows:

We are among the first to consider multiple vehicle cooperative localization with unknown biases under Random Finite Set Theory. The proposed PHD filter can estimate and compensate for the measurement biases accurately, which performs better localizations. In addition, by utilizing the PHD filter, the challenges for multiple vehicle cooperative localization are also overcome [18], e.g., the communication bandwidth is bounded and the data association issue is eliminated.

The rest of this paper is organized as follows: Section 2 briefly describes multiple vehicle cooperative localization with measurement misregistration. Section 3 introduces the mathematic background of the PHD and its implementation. Section 4 presents simulation results. Finally, the paper is concluded in Section 5.

## 2. Background Description

**Figure 1.**
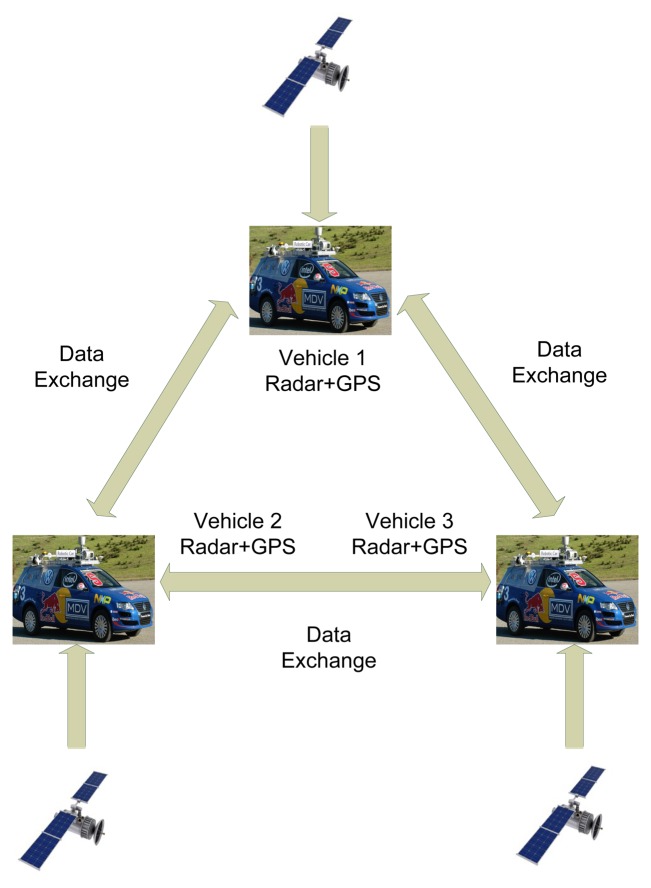
Multiple vehicle cooperative localization system.

As illustrated in Figure 1, the description of multiple vehicle cooperative localization is as follows:

- Each vehicle is able to localize itself. Here, we assume that the corresponding measurements are in the 2D global coordinate.
- Each vehicle is able to measure the relative position of the other vehicles. Here, we assume that the measurements are formatted with the range and bearing in a local coordinate. Furthermore, each measurement error is formed of white noises and fixed biases.
- Vehicles are equipped with communication transceivers for information exchanging.
- The communication network does not have the identification capability regarding the data association issue. Each vehicle observes the whole environment and transmits its observations over the network. There is no prior information regarding the data association issue provided by the inter-vehicle communication.

Multiple vehicle cooperative localization improves the precision of the localization. Assuming the measurements' noises for proprioceptive sensors are larger than a certain threshold, the localization may be imprecise when vehicles are aggregated in a small region. However, the precision is ensured with the help of the exteroceptive sensors. Furthermore, vehicles can localize themselves under the condition that only one proprioceptive sensor is available, with the help of cooperative localization.

Much work has been done for cooperative localization by using a centralized extended Kalman filter [10,12] and a decentralized solution [11,25,26]. The state of the group is viewed as a single system. The localization is obtained by exchanged data from each vehicle. The Kalman filter reduces the uncertainty of the localization by information sharing. However, the disadvantage is the large quantity of transmitted data. This quantity grows exponentially as the number of vehicles increases. In addition, for a dynamic multiple vehicle structure, the Kalman filter requires an association process, which takes huge resources.

Instead of a centralized architecture, a decentralized solution is investigated, where multiple fusion centers exist and each of them handles only local information (only the observed neighbors). However, the computational demand is very high. In addition, it often leads to the over-convergence problem when handling inter-estimate correlation among various sources. Since the over-convergence problem is caused by inter-estimation correlation, a natural idea for addressing this problem is investigated controlling of the data flow within the vehicle network. Howard [13] maintains a heuristic method based on a dependency tree to update the distribution dependencies. However, the author assumes that distributions are only dependent on the distribution that was last used and are independent of the others. This assumption is restrictive, as circular updates can still occur. The authors in [25] propose a state exchange-based method, which only allows independent estimates to be shared within the network. However, communication and computation demand is also increased. The authors in [26] proposed a covariance intersection filter [27] to handle the inter-estimate correlation issue. Similar work can also be found in [28,29], which exploits the advantage of estimation. However, as mentioned above, the computational complexity becomes an issue when the number of vehicles increases.

**Figure 2.**
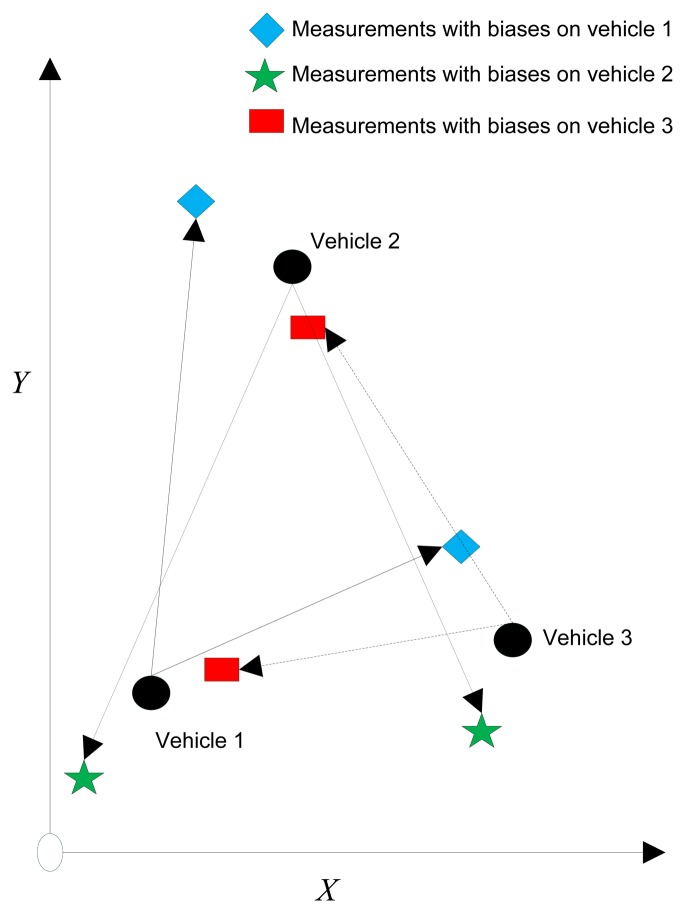
Measurements from the exteroceptive sensor.

Neither centralized approaches nor decentralized approaches consider the spatial registration issue during the localization process. As we can see from Figure 2, each vehicle observes its surrounding environment with the help of the exteroceptive sensors. However, since the biases exist, the network cannot localize well when the local sensor coordinate transformed to the global coordinate. Therefore, the goal of multiple vehicle cooperative localization is to take spatial registration into account to acquire a more precise localization.

In this paper, we adopt the sequential Monte Carlo PHD filter implementation to jointly estimate the biases and the states simultaneously. The proposed SMC PHD filter handles spatial registration well under multiple vehicle cooperative scenarios during the whole process.

## 3. The Probability Hypothesis Density Filter

The PHD filter based on Random Finite Random Finite Set Theory is proposed because of its superior performance in the multiple targets tracking domain.

### 3.1. Overview on RFS Statistics

**Figure 3.**
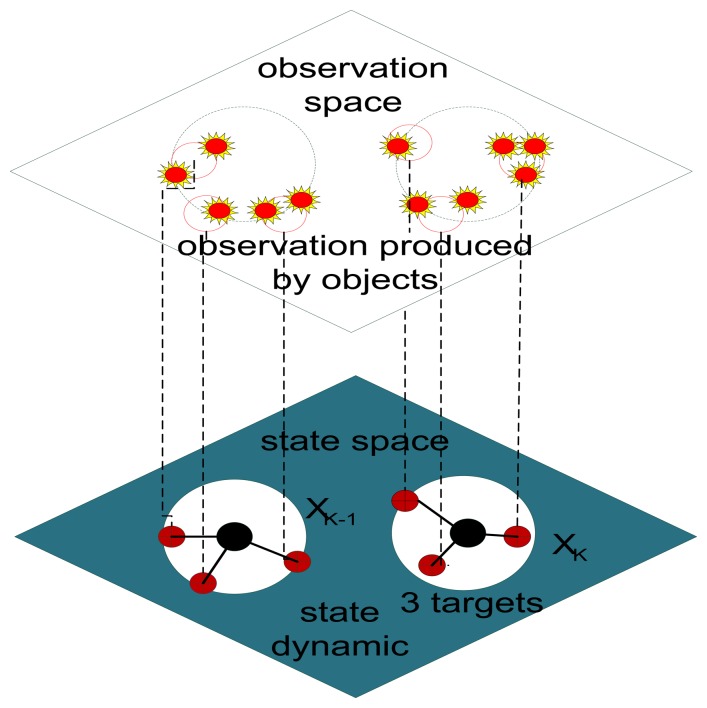
Set-valued states and set-valued observations.

The random finite set is a hidden Markov chain model with set-valued state and set-valued observation, while the PHD filter is a predicted and corrected framework for recursive Bayesian filtering in such an RFS formulation. A comparison of the RFS approach and traditional multiple-target tracking methods has been given in [30]. In the PHD filter, the collection of individual targets is treated as a set-valued state, and the collection of individual observations is treated as a set-valued observation. Figure 3 is a basic introduction to the PHD filter, which illustrates that the observations and the corresponding states are considered on a single-valued space at each step [31]. The PHD filter operates on the set space and avoids the combinatorial problem that arises from data association.

Regarding the superior performance, the PHD filter has been used for various scenarios, e.g., SLAM [32,33], extended target tracking [34] and multi-robot localization [35,36].

This paper extends the earlier work for multiple vehicle cooperative localization [18] by utilizing the sequential Monte Carlo PHD solution. The sequential Monte Carlo PHD implementation for biases estimation is first proposed by Lian *et al.* in [24]. In this paper, the multiple vehicle localization scenario is considered to exhibit its great potentials.

### 3.2. Mathematic Background

The targets in a multi-target scenario at time *k* are represented as a finite set of vectors, X*_k_*_,1_,…, X*_kN_*_(_*_k_*_)_, which takes values from the state space, *χ* ∈ ℝ*^n^*^x^.

(1) $$X_{k}=\{x_{k,1},\ldots ,x_{k,N(k)}\}\in ℱ(\chi )$$

where *N*(*k*) represents the number of targets at time *k* and *ℱ*(*χ*) denotes the set of all finite subsets of *χ*. The dynamics of the existing target is modeled by a Markov process with transition density *f_k_*_|_*_k_*_− 1_(x*_k_*|x*_k_*_−1_).

Similarly, the observations are represented as a finite set of vectors. Suppose *L* sensors with overlapping coverage provide measurements of the targets in *χ* by taking values in the observation space, *Ƶ* ∈ ℝ*^n^*^z^.

(2) $$Z_{k}^{l}=\{z_{k,1}^{l},\ldots ,z_{k,M(k)}^{l}\}\in ℱ(\chi )$$

where *M*(*k*) represents the number of observations at time *k* and *ℱ*(*Ƶ*) denotes the set of all finite subsets of *Ƶ*. Measurements are affected by two sources of error: the systematic errors (biases; and the stochastic zero mean additive noise. For measurement $z_{k}^{l}\in Z_{k}^{l}$, which originates from target x*_k_* ∈ *X_k_*, the measurement equation can be expressed as:

(3) $$z_{k}^{l}=h_{k}^{l}(x_{k})+\beta_{k}^{l}+v_{k}^{l}$$

where $h_{k}^{l}$ is the nonlinear measurement function of sensor *l*; $\beta_{k}^{l}$ is the bias vector of sensor *l* and $v_{k}^{l}$ is zero mean Gaussian noise.

Let $\beta_{k}={\left[{\left(\beta_{k}^{1}\right)}^{T},\ldots ,{\left(\beta_{k}^{L}\right)}^{T}\right]}^{T}$ denote the *L* sensors' measurement bias vector; the task is to estimate the posterior PDF of the joint states *X_k_* and bias *β_k_*. This posterior is denoted by *p_k_*(*X_k_*, *β_k_*|*Z*_1:_*_k_*), where *Z*_1:_*_k_* is a shortened notation for the sequence of measurement sets received up to time *k*. With the goal of deriving the PHD filter for joint spatial registration and states estimation, joint state **y***_k_* = (**x***_k_*, *β_k_*) is therefore augmented.

Using these random finite set models, it is possible to construct process and observation models analogous to the single-target case. Randomness in *X_k_*, *β_k_* and *Z_k_* can be encapsulated into a multi-target transition density and multi-target observation likelihood.

Under the above models, the multi-target Bayes filter propagates the posterior multi-target density, *π_k_*(·|*Z*_1:_*_k_*), recursively in time. However, due to its combinatorial nature, it is intractable in most applications. To alleviate this, the PHD filter propagates the first moment or PHD *D_k_*(·) of multi-target posterior density *π_k_*(·).

The PHD recursion is given by:

(4) $$D_{k|k-1}(y_{k})=\int P_{S,k}(y_{k-1})f_{k|k-1}(y_{k}|y_{k-1})D_{k-1}(y_{k-1})dy_{k-1}+\gamma_{k}(y_{k})$$

(5) $$D_{k}({\text{y}}_{k})=(1-P_{D,k}(y_{k}))D_{k|k-1}(y_{k})+\sum_{z\in Z_{k}}\frac{P_{D,k}(y_{k})g_{k}(z|y_{k})D_{k|k-1}(y_{k})}{\kappa_{k}(z)+\int P_{D,k}g_{k}(z|\zeta )D_{k|k-1}(\zeta )d\zeta }$$

where *P_D,k_* denotes the probability that the measurements originate from the targets and *P_S,k_* denotes the probability that the target survives at time *k*. The intensity function, *γ_k_*(·), illustrates the birth target between *k* − 1 and *k*, whereas the clutter is specified by its intensity, *κ_k_*(·).

Analogous to the standard PHD filter, the spawn process of the augmented state is modeled as Poisson RFS with intensity *ρ*(**y***_k_*|**y***_k_*_−1_). Assuming the state, *X_k_*, is independent of bias *β_k_*, the birth and spawn intensities, the transition density and the survival probability of the joint state, **y***_k_*, are written as:

(6) $$\begin{array}{c} \gamma_{k}({\text{y}}_{k})=\gamma_{k}(x_{k})+\gamma_{k}(\beta_{k})\\ \rho_{k}({\text{y}}_{k}|y_{k-1})=\rho (x_{k}|x_{k-1})+\rho (\beta_{k}|\beta_{k-1})\\ f_{k|k-1}(y_{k}|y_{k-1})=f_{x,k|k-1}(x_{k}|x_{k-1})f_{\beta ,k|k-1}(\beta_{k}|\beta_{k-1})\\ P_{S,k}(y_{k-1})=P_{S,k}(x_{k-1})P_{S,k}(\beta_{k-1}) \end{array}$$

Since the sensor biases are considered as non-random parameters, the following equations are therefore acquired:

(7) $$\begin{array}{ccc} \gamma_{k}(\beta_{k})=0, & \rho (\beta_{k}|\beta_{k-1})=0, & P_{S,k}(\beta_{k-1})=1 \end{array}$$

while the PHD recursion is therefore revised by:

(8) $$\begin{array}{c} D_{k|k-1}(y_{k})=D_{k|k-1}({\text{x}}_{k},\beta_{k}|Z_{1:k-1}^{l:L})\\ =\int \left[P_{S,k}({\text{x}}_{k-1})f_{x,k|k-1}(x_{k}|x_{k-1})f_{\beta ,k|k-1}(\beta_{k}|\beta_{k-1})+\rho (x_{k}|x_{k-1})\right]\\ \cdot D_{k-1}(x_{k-1},\beta_{k-1}|Z_{1:k-1}^{l:L})dx_{k-1}d\beta_{k-1}+\gamma_{k}(x_{k}) \end{array}$$

Assume the biases are independent; the PHD update step is approximated as:

(9) $$\begin{array}{c} D_{k|k}(y_{k})=D_{k|k}({\text{x}}_{k},\beta_{k}|Z_{1:k}^{l:L})\\ =G_{k}^{1}(Z_{k}^{1}|{\text{x}}_{k},\beta_{k}^{1})\cdots G_{k}^{L}(Z_{k}^{L}|x_{k},\beta_{k}^{L})\cdot D_{k|k-1}(x_{k},\beta_{k}|Z_{1:k-1}^{l:L}) \end{array}$$

where:

(10) $$\begin{array}{c} G_{k}^{l}(Z_{k}^{l}|{\text{x}}_{k},\beta_{k}^{l})=1-P_{D,k}^{l}(x_{k},\beta_{k}^{l})\\ +\sum_{z_{k}^{l}\in {Z_{k}}_{l}^{k}}\frac{P_{D,k}^{l}(x_{k},\beta_{k}^{l})g_{k}^{l}(z_{k}^{l}|x_{k},\beta_{k}^{l})}{k_{k}^{l}(z_{k}^{l})+\int P_{D,k}^{l}(x_{k},\beta_{k}^{l})g_{k}^{l}(z_{k}^{l}|x_{k},\beta_{k}^{l})D_{k|k-1}(x_{k},\beta_{k}|Z_{1:k-1}^{l:L})dx_{k}d\beta_{k}} \end{array}$$

The integral of the proposed PHD recursion on region *S* of the joint state, **y***_k_*, is defined as the expected number of the objects in *S*:

(11) $${\hat{N}}_{k|k}=\int D_{k|k}(y_{k})dy_{k}=\int D_{k|k}(x_{k},\beta_{k}|Z_{1:k}^{l:L})dx_{k}d\beta_{k}$$

while the biases are therefore derived by:

(12) $${\hat{\beta }}_{k|k}=\frac{\int_{S}\beta_{k}D_{k|k}(x_{k},\beta_{k}|Z_{1:k}^{l:L})dx_{k}d\beta_{k}}{{\hat{N}}_{k|k}}$$

It is noted that the PHD recursion has multiple integrals that have no closed form solutions in general. Therefore, the sequential Monte Carlo method is implemented. The MCMC (Markov Chain Monte Carlo) move step is provided for increasing the particle variety after the re-sample step, without affecting the validity of the approximation [37]. The K-means method is also utilized to cluster the resampled particles [38]. More details of the SMC PHD implementation can be found in [24]. Based on the above procedure, we address the issues for the PHD filter in multiple vehicle cooperative localization with spatial registration. The exteroceptive sensors' biases (bearing and range) and each vehicles' location are estimated simultaneously during the whole process.

### 3.3. Implementation Issues

For the process model, the state **x***_k_* = [*p_x,k_*, *p_y,k_*, *ṗ_x,k_*, *ṗ_y,k_*]*^T^* of each vehicle consists of position (*p_x,k_*,*p_y,k_*) and velocity (*ṗ_x,k_*, *ṗ_y,k_*). Assume that the process noise is zero mean Gaussian white noise with the covariance matrix, *Q_k_*. Then, the Markovian transition probability density of x*_k_* is modeled as:

(13) $$f_{k|k-1}(x|\zeta )=N(x;F_{k-1}\zeta ,Q_{k-1})$$

where *Q_k_* and *F_k_* are given by the constant velocity model as:

(14) $$\begin{array}{cc} F_{k}=\left[\begin{array}{cc} {\text{I}}_{2} & {\text{I}}_{2}\\ 0_{2} & I_{2} \end{array}\right], & Q_{k}=\delta^{2}\left[\begin{array}{cc} I_{2}/4 & I_{2}/2\\ 0_{2} & I_{2} \end{array}\right] \end{array}$$

where **I***_n_* and **O***_n_* denote, respectively, the *n*×*n* identity and zero matrices and *δ* is the standard deviation of the process noise.

For the measurement model, measurements are originated from both proprioceptive and exteroceptive sensors projecting to the ground plane. To map the state to the observation space, the observation matrix is *H_k_* = [**I**_2_, **0**_2_], while the measurement vector is **z** = [*p_x_*,*p_y_*]*^T^*.

The measurement errors of the exteroceptive sensors are comprised of the biases $\beta_{k}^{l}={\left[\Delta \rho_{k}^{l},\Delta \theta_{k}^{l}\right]}^{T}$ (range and bearing) and the random measurement noises $v_{k}^{l}={\left[\delta \rho_{k}^{l},\delta \theta_{k}^{l}\right]}^{T}$. Assume that the biases do not drift against time; the dynamic model $\beta_{k}={\left[{\left(\beta_{k}^{1}\right)}^{\text{T}},\ldots ,{\left(\beta_{k}^{L}\right)}^{T}\right]}^{T}$ can be considered as a first order Gauss–Markov process with the transition density:

$$f_{\beta ,k|k-1}(\beta_{k}|\beta_{k-1})=N(\beta_{k}^{1}|\beta_{k-1}^{1},B_{k-1}^{1})\cdots \times N(\beta_{k}^{L}|\beta_{k-1}^{L},B_{k-1}^{L})$$

Here, we only consider the measurements from both proprioceptive and exteroceptive sensors as the set-valued observation. For instance, we measure the vehicles' relative positions according to the exteroceptive sensors. Measurements are then acquired by coordinate transformation to the global plane. Assuming there are *L* vehicles for the cooperative localization, the size of the observation set is *L*(*L* − 1) at each step.

## 4. Simulation

The simulation was implemented on the ground plane over the surveillance region [−6, 000, 6, 000] × [−6,000,6,000] m^2^ for a period of 200 s. In fact, with the number of vehicles increasing, the final results are affected, due to the uncertainty issues. In this paper, only four vehicles are implemented to illustrate the potential of the PHD filter in the field of cooperative localization. In simulation, the biases of the exteroceptive sensors are considered as *β*^1^ = [−25 m, 75 mrad]*^T^*, *β*^2^ = [55 m, −60 mrad]*^T^*, *β*^3^ = [−40 m, 25 mrad]*^T^*, *β*^4^ = [35 m, −45 mrad]*^T^*. The random measurement noise for the proprioceptive sensor is assumed to be zero mean Gaussian white noise with covariance matrix R*_GPS_* = diag[15 m^2^,15 m^2^], while the exteroceptive sensor is with R*_Radar_* = diag[5 m^2^, 5 mrad^2^]. The inter-vehicle communication is also available to exchange the information on the network. In addition, the V2V communication system does not have the ability to identify the others during the whole process. For the purpose of comparison, the simulation is also compared with the standard PHD filter, which does not consider the spatial registration [18].

**Figure 4.**
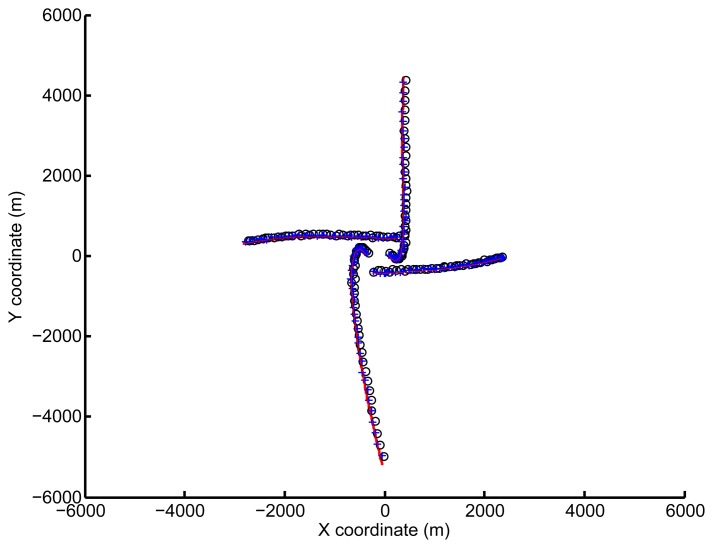
Estimated positions by both filters.

In Figure 4, “+” denotes the vehicle position estimated by the PHD filter after considering spatial registration, while “○” denotes the standard PHD estimation. The solid line denotes the actual vehicle trajectory. The whole trajectory of each vehicle is missing, due to the PHD filter. Compared with standard target tracking methods, the PHD operates on the set space and avoids the association issue. Both observations and estimations are set-valued (for example, although the PHD filter can estimate the number of the vehicles and the corresponding states, it does not distinguish between them. Thus, the identification of a single vehicle over the whole process is not available;. However, for multiple vehicle cooperative localization, keeping the whole trajectory of each vehicle is out of the scope of this paper.

It can be seen that the estimation derived by the standard PHD filter deviates from the truth, due to the the effect of the sensor bias. However, after considering the spatial registration, the estimations are more close to the true trajectories.

Furthermore, the circular position error probability (CPEP) is also utilized to evaluate the performance of both methods. Given the true and estimated state sets $\chi_{k}={\left\{x_{k}^{l}\right\}}_{l=1}^{L_{k}}$ and ${\hat{\chi }}_{k|k}={\left\{{\hat{x}}_{k|k}^{l}\right\}}_{l=1}^{{\hat{L}}_{k|k}}$, the CPEP is calculated by:

(15) $${\text{CPEP}}_{k}(r)=\begin{array}{cc} \frac{1}{L_{k}}\sum_{x_{k}\in \chi_{k}}\text{Prob}\{{\left\|H_{k}{\hat{x}}_{k|k}-H_{k}x_{k}\right\|}_{2}>r, & \forall {\hat{x}}_{k|k}\in {\hat{\chi }}_{k|k}\} \end{array}$$

where *r* is the radius of CPEP. *H_k_***x̂***_k_*_|_*_k_* and *H_k_***x***_k_* denote the estimated and true positions in global coordinates. In this paper, we let *r* = 20 m.

**Figure 5.**
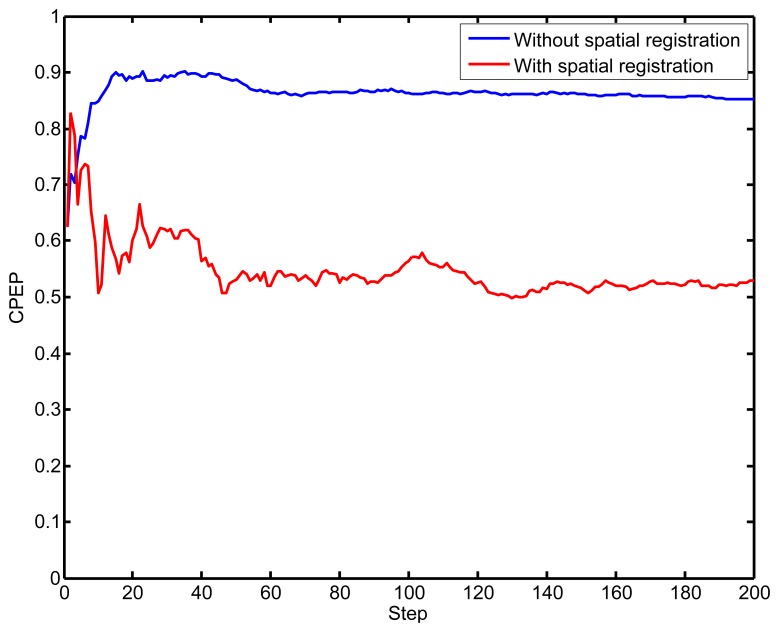
The circular position error probability (CPEP) against time.

Figure 5 exhibits the CPEP at each step. It illustrates that the CPEP is smaller than the standard PHD by considering the sensors' biases, which exhibits the conclusion that cooperative localization with spatial registration is more close to the ground truth. The standard PHD filter's performance is rather ineffective without considering the spatial registration issue.

**Figure 6.**
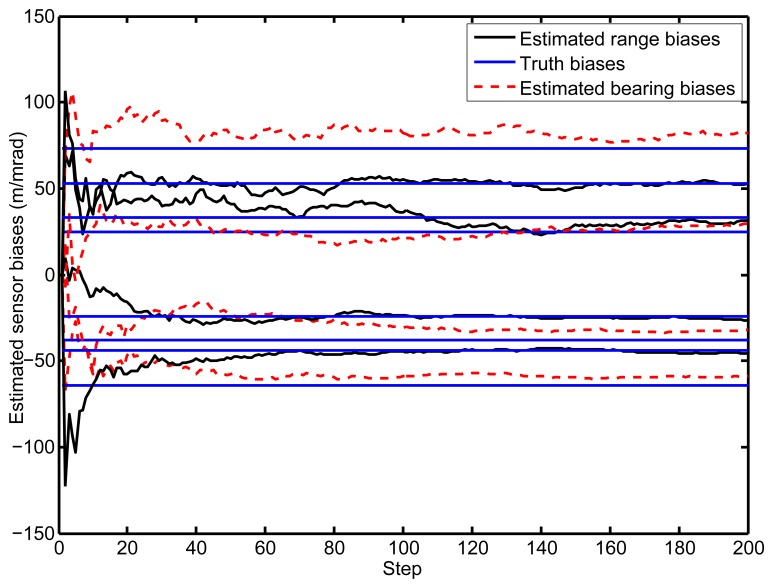
The estimated biases of the vehicles.

Figure 6 illustrates the estimations of the biases during the whole process. As we can see, the estimated values converge to the true biases in a few steps. After the biases elimination in the proposed PHD filter, the cooperative localization accuracy is significantly improved compared with the standard PHD filter, which is exhibited in Figure 5.

**Figure 7.**
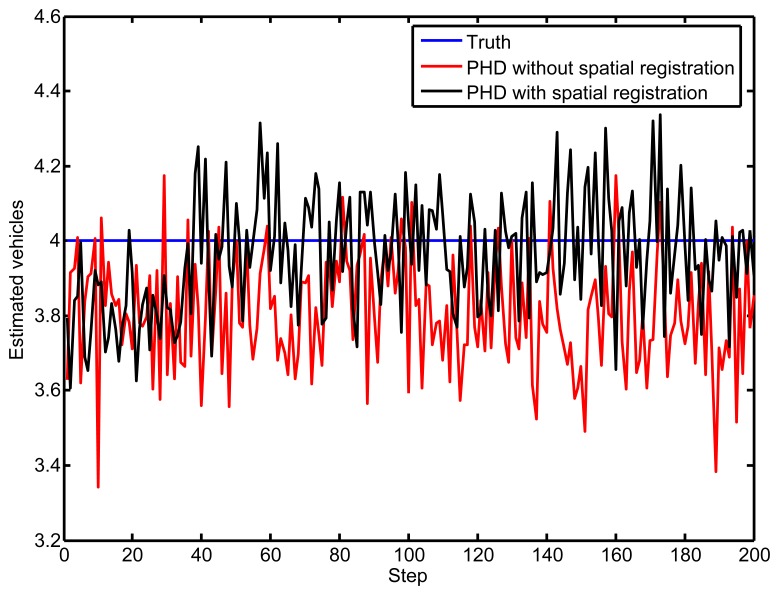
The estimated number of vehicles. PHD, probability hypothesis density.

Figure 7 illustrates the number of vehicles, which is estimated by the PHD filter under the data association uncertainty issue. With time increasing, the vehicle numbers estimated by the proposed PHD filter are close to the true number, by the process of bias elimination. However, the performance of the standard PHD filter is imprecise compared to the true vehicle number, without eliminating the sensors' biases.

In conclusion, combined with our previous work [18], the benefits of the PHD filter are as follows:

First, it reduces and bounds the requirements of the communication bandwidth in a multiple vehicle environment. The inter-vehicle communication system transmits the measurements to the PHD filter, which takes little bandwidth. Compared to other methods, the proposed approach has the lowest consumption requirements for the communication bandwidth, since each vehicle only transmits its observations.

Second, it works under extreme conditions, which often happen in real environments (where the association uncertainty exists, the number of the vehicles is unknown, sensor delays and communication unavailability occur and the measurement is distracted by noise). The PHD filter not only illustrates the high performance of the localization, but also exhibits the robustness under the dynamic structure of the group.

Third, the spatial registration issue is addressed to jointly estimate the states and the corresponding biases during the whole process. Compared to others, the concept of multiple vehicle cooperative localization with spatial registration is first proposed under Random Finite Set Theory.

Because of the superiority mentioned above, we have strong reason to believe that the PHD filter has a great potential in the field of cooperative localization.

## 5. Conclusions

In this paper, a recursive Bayesian solution for multiple vehicle cooperative localization with spatial registration is first proposed. The sensors' biases and the vehicles' states are estimated simultaneously based on the PHD filter. In comparison to related work, the whole group of vehicles and the sensors' biases are viewed as a single set-valued state, the measurements are collected as a single set-valued observation, which is used to update the behavior of the set-valued state. The proposed approach also overcomes the challenges existing in multiple vehicle cooperative localization, e.g., low communication bandwidth and data association uncertainty. Experimental results exhibit the high performance of the joint spatial registration PHD filter.

Future work will focus on the evaluation of the proposed approach in a non-synthetic environment.
